# Citrullinated and MMP-degraded vimentin is associated with chronic pulmonary diseases and genetic variants in PADI3/PADI4 and CFH in postmenopausal women

**DOI:** 10.1038/s41598-023-50313-y

**Published:** 2023-12-27

**Authors:** Cecilie Liv Bager, Joseph P. M. Blair, Man-Hung Eric Tang, Joachim Høg Mortensen, Anne-Christine Bay-Jensen, Peder Frederiksen, Diana Leeming, Claus Christiansen, Morten Asser Karsdal

**Affiliations:** Nordic Bioscience, Biomarkers and Research, Hovedgade 205-207, 2730 Herlev, Denmark

**Keywords:** Immunology, Biomarkers, Pathogenesis, Respiratory tract diseases

## Abstract

Citrullinated vimentin has been linked to several chronic and autoimmune diseases, but how citrullinated vimentin is associated with disease prevalence and genetic variants in a clinical setting remains unknown. The aim of this study was to obtain a better understanding of the genetic variants and pathologies associated with citrullinated and MMP-degraded vimentin. Patient Registry data, serum samples and genotypes were collected for a total of 4369 Danish post-menopausal women enrolled in the Prospective Epidemiologic and Risk Factor study (PERF). Circulating citrullinated and MMP-degraded vimentin (VICM) was measured. Genome-wide association studies (GWAS) and phenome wide association studies (PheWAS) with levels of VICM were performed. High levels of VICM were significantly associated with the prevalence of chronic pulmonary diseases and death from respiratory and cardiovascular diseases (CVD). GWAS identified 33 single nucleotide polymorphisms (SNPs) with a significant association with VICM. These variants were in the peptidylarginine deiminase 3/4 (PADI3/PADI4) and Complement Factor H (CFH)/KCNT2 gene loci on chromosome 1. Serum levels of VICM, a marker of citrullinated and MMP-degraded vimentin, were associated with chronic pulmonary diseases and genetic variance in PADI3/PADI4 and CFH/ KCNT2. This points to the potential for VICM to be used as an activity marker of both citrullination and inflammation, identifying responders to targeted treatment and patients likely to experience disease progression.

## Introduction

During cellular stress, proteins can be post-translationally modified enabling them to be recognized by the immune system. One such type of stress-induced posttranslational modification is citrullination catalyzed by peptidylarginine deiminase (PAD) enzymes. In rheumatoid arthritis (RA) anti-citrullinated protein antibodies (ACPA) provide both diagnostic and prognostic value. More recently, however, citrullination has been shown to be a central part of immune activation, not only in RA, but in a wide range of chronic diseases such as chronic obstructive pulmonary disease (COPD)^1^, inflammatory bowel disease (IBD)^2^ and cancer^3^. When dysregulated, citrullination becomes a common denominator in these chronic diseases, however it has not yet been assessed which pathways and diseases are linked with citrullinated peptides in a large clinical setting.

One protein that is known to undergo citrullination is the intermediate filament protein vimentin^4^. Vimentin knockout, or inhibition of vimentin expression leads to decreased collagen production and protection from development of pulmonary fibrosis^5–7^. Furthermore, treatment with citrullinated vimentin mediates development and progression of lung fibrosis through Toll-Like Receptor 4 (TLR4)-dependent nuclear factor kappa-light-chain-enhancer of activated B cells (NF-κB) activation in mice^8^.

The VICM biomarker can assess a citrullinated and MMP-degraded fragment of vimentin that is released from activated macrophages. Previous studies have shown that VICM reflects disease burden in ankylosing spondylitis^9^ and is associated with treatment response in RA patients^10^. Elevated VICM has also previously been associated with patients with COPD and elevated in patients with progressive Idiopathic Pulmonary disease (IPF)^11^. Finally, VICM also has implications in IBD, being able to differentiate ulcerative colitis and Crohn’s disease^12^.

These results suggest that MMP degradation of citrullinated vimentin is associated with several chronic diseases. How these processes drive disease progression in different chronic inflammatory diseases remains unclear.

The aim of this study was to obtain a better understanding of the genetic variants and pathologies associated with citrullinated and MMP-degraded vimentin. In this paper we conducted a genome wide association study (GWAS) and phenome wide association study (PheWAS) for the VICM biomarker using the Prospective Epidemiologic Risk Factor (PERF) study^13^, an observational cohort with 5855 women linked to the Danish health registries.

## Materials and methods

### Study design

PERF is comprised of 5855 post-menopausal Danish women, enrolled at baseline (PERF I) between 1999 and 2001. The aim of the study was to identify potential risk factors associated with age-related diseases. All participants had previously been enrolled in randomized placebo-controlled trials or had been screened for previous studies in Copenhagen or Aalborg at the Center for Clinical and Basic Research^13^.

The study was carried out in accordance with Good Clinical Practice (ICH-GCP) and Declaration of Helsinki. The study protocol was approved by the Danish Research Ethics Committee (KA99070gm). Informed consent was obtained from all participants or legal guardians.

Patients were excluded from the study based on missing genotype data and filtered based on a population level genotype filter to remove participants with cryptic relatedness. Patients without registry data were excluded from the study, as were patients with missing VICM measurements.

### Data collection

As part of the PERF study, participants were interviewed by a doctor or nurse at baseline, allowing collection of data pertaining to demographics, lifestyle, and medical history. For those who consented, fasting serum (n = 5668) and DNA samples were collected (n = 5553).

VICM (commercial name: nordicVICM™, catalogue number: 1800AG01, vendor: Nordic Bioscience, Herlev, Denmark) was measured blinded in serum by enzyme-linked immunosorbent assay (ELISA) in a CAP-certified laboratory as previously described^14^. Lymphocyte and neutrophil counts were determined using an automated blood cell analyser (Sysmex).

Disease history from each consenting participant (n = 5602) was collected from the Danish National Patient Registry (NPR), made possible by linking each participant’s civil registration number (CPR number) to the NPR. Data was collected for the period 1974–2014 and was censored on 31-12-2014, corresponding to the end of PERF study.

Disease phenotypes used in this study were classified through a use of the NPR, biomarker measurements and questionnaire data (Supplementary Table S1). Phenotypes were defined as pre-baseline plus one year, and all-time occurrence.

### Genotyping

A total of 5516 samples were successfully genotyped of the 5553 samples available. Genotyping was then performed using a custom-made Illumina Global Screening Array version 2 (693143 probes) in collaboration with deCODE Genetics Iceland.

### Probe and individual filtering

Standard probe-level filtering was performed, using a minor allele frequency of greater than or equal to 1%, a Hardy Weinberg Equilibrium p-value cutoff greater than or equal to 1e−6, and a minimum probe call rate of 97%. No multi-allelic SNP filtering was conducted.

A total of 534710 probes were screened for association to serological VICM levels. Identity-by-descent and the inbreeding coefficient were calculated using the Plink -genome and -ibc functions respectively, to address cryptic relatedness. Study participants were removed on a one-side-of-a-pair basis, using a pi_hat = 0.1875 cut-off value. A cut off value of less than − 0.1 of or greater than 0.1 was used for the Fhat2 coefficient. Of the 5516 available genotyped study participants, 136 were removed.

### Principal component analysis

EIGENSTRAT Smartpca 7.2.0^15^ was used to conduct principal component analysis (PCA) of the genotypes in the available population (n = 5106) for all 534,710 filtered variants. 10 principal components were extracted with 5 iterations used. The 3 leading components capture 0.3% of the explained variance.

### Linear regression

Using plink v1.90p^16^ linear additive regression was conducted on the study population (n = 4369), whilst adjusting for age at baseline, BMI, and the leading three principal components. Plink switches—allow-no-sex to allow samples with missing gender information and—keep-allele-order to assign allele A1 to the ALT allele were used.

GWAS significance thresholds were defined as equal to 5e−8 for the genome-wide significance threshold, and 1e−5 for the suggestive significance threshold. P-values were visualized in a Manhattan plot, using the R package qqman^17^.

### Pathway analysis/enrichment

Following GWAS analysis, pathway enrichment analysis was performed using VEGAS2^18^ and PARIS 2.4^19^ using default parameters. VEGAS2 analysis was performed using the Biosystems gene/pathway annotation file provided by the software website. The LOKI knowledge base used by PARIS2 was compiled in February 2020. Significant associations to REACTOME pathways which were common to both frameworks were reported, using p < 0.05.

### Statistical analysis

Logistic regression was performed between serum levels of VICM and disease phenotypes, whilst adjusting for age, BMI, and smoking. P-values were corrected for multiple hypothesis testing using FDR, with a threshold of 0.05. Cause specific Cox proportional hazards regression analyses were performed between VICM and major causes of death, with cause of death being taken from the patient death registry. Age was used as the time scale, and the models were adjusted for BMI and smoking. All analysis was done in R version 3.6.0^20^ with plotting done with cox models built using rms^21^ and plotted using ggplot2^22^.

## Results

### Study design

At baseline, 5855 patients were included in the PERF cohort. Of these, 4369 patients were identified suitable to be included in the study, with available serological measurements, genotype data, disease history and cause of death from the NPR. Key demographics and clinical characteristics of the patients can be seen in Table 1. Occurrences of each disease phenotype are shown in Supplementary Table S1 and definitions in Supplementary Table S2.

**Table 1:** Overview of the study population used to investigate genetic and phenotypic traits associated to levels of the VICM biomarker.

Clinical characteristic	
Population size	4369
Baseline age (mean (Sd))	70.04 (6.52)
BMI (mean (Sd))	26.14 (4.21)
Diastolic Bp (mean (Sd))	81.75 (11.40)
Systolic Bp (mean (Sd))	149.98 (24.23)
VICM (mean (Sd))	1.83 (1.16)
Smoking (n(%))	
Never	2075 (47.5)
Former	1347 (30.8)
Current	947 (21.7)
Neutrophil–lymphocyte ratio (n(%))	
NLR < 3	3651 (88.3)
3 ≤ NLR < 5	430 (10.4)
NLR ≥ 5	53 (1.3)
NA	235 (5.4)

*BP* blood pressure, *NLR* neutrophil–lymphocyte ratio, *SD* standard deviation, *VICM* citrullinated and MMP degraded vimentin.

### Relation of VICM to baseline characteristics

**Figure 1:**
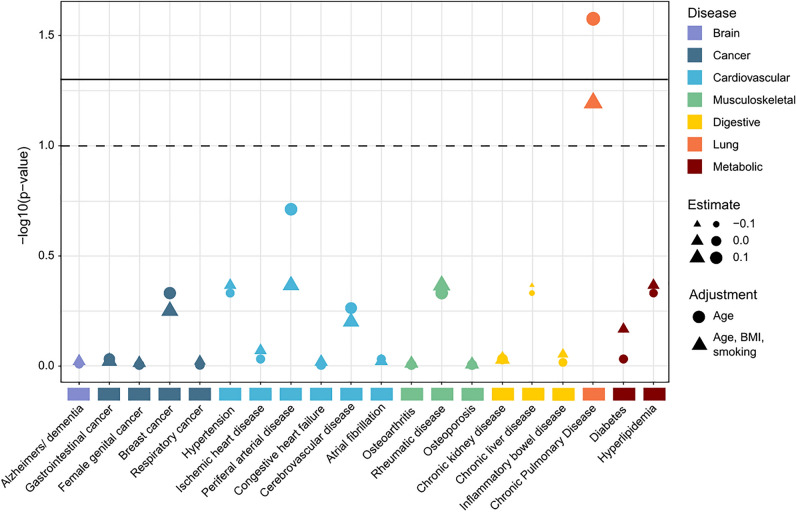
Association of pre-baseline disease phenotypes with baseline VICM levels identifies a significant relationship between high levels of VICM and chronic pulmonary disease.

Association between levels of baseline VICM and predefined disease phenotypes prior to baseline, or up to 1 year after, was explored. FDR corrected p-values for each phenotype from logistic regression modeling corrected for age, BMI and smoking in the adjusted model, and age alone in the non-adjusted model can be seen in Fig. 1. We observed that VICM levels were significantly associated with chronic pulmonary disease when adjusting for age. When adjusting for age, BMI and smoking status we did however not see a significant association after FDR correction.

Further associations were investigated with respect to VICM and smoking and neutrophil–lymphocyte ratio (NLR). Supplementary Fig. S1A shows the relationship between NLR and VICM. We can see that those patients with high NLR (NLR > 5) also have significantly higher levels of VICM (ANOVA, p = 2.2e−16). Supplementary Fig. S1B, we can see that smoking is significantly associated with smoking status (ANOVA, p = 1.6e−10). A Tukey post-hoc test revealed that current smokers had a significantly higher VICM level than those patients who had previously smoked or never smoked (p < 0.001), whilst non-smokers and former smokers showed no statistical difference.

### VICM and its association to death

**Figure 2:**
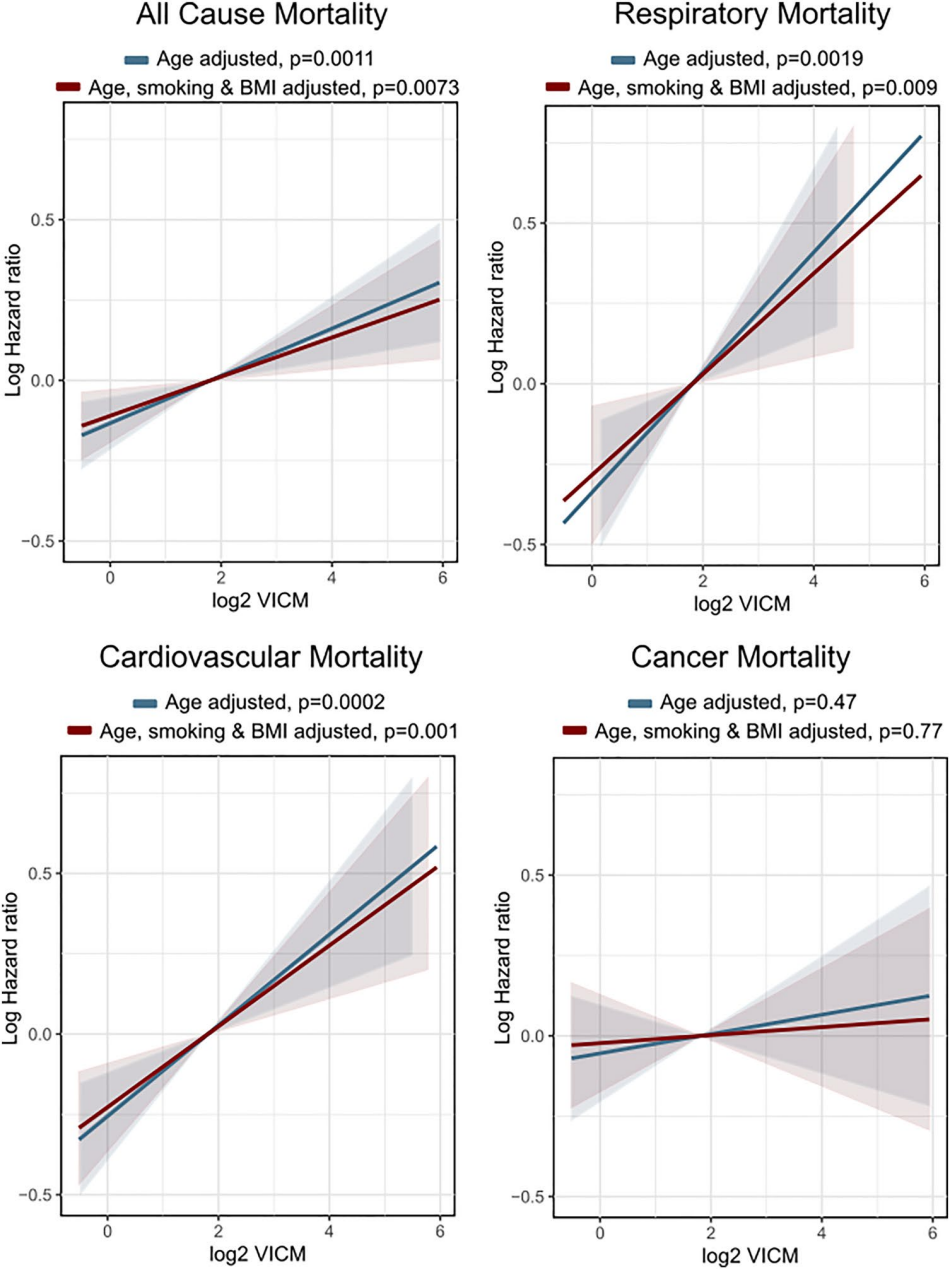
Estimated hazard ratios of all-cause mortality by levels of VICM, cause-specific hazard ratios of death due to respiratory disease, CVD, and cancer by levels of VICM. Blue lines are adjusted for age and red lines are adjusted for age, smoking and BMI. Specific hazards can be seen in Supplementary Table S3.

Baseline levels of VICM were associated with time to death, relative to age. Causes of death investigated were all-cause mortality, causes related to respiratory disease, cardiovascular disease, and malignant cancers, the top three causes of mortality in PERF. These associations were assessed in cause-specific Cox proportional hazards models, whilst controlling for BMI and smoking. We observed that higher levels of VICM were associated with higher hazard of death with all-cause mortality. This was also true for hazards of death from respiratory disease or cardiovascular disease, both significantly associated with increased levels of VICM. The cause-specific hazard of death due malignant cancer was not significantly associated with VICM levels. These associations can be seen in Fig. 2, with hazard ratios reported in Supplementary Table S3.

### Genome wide associations with serological VICM

**Figure 3:**
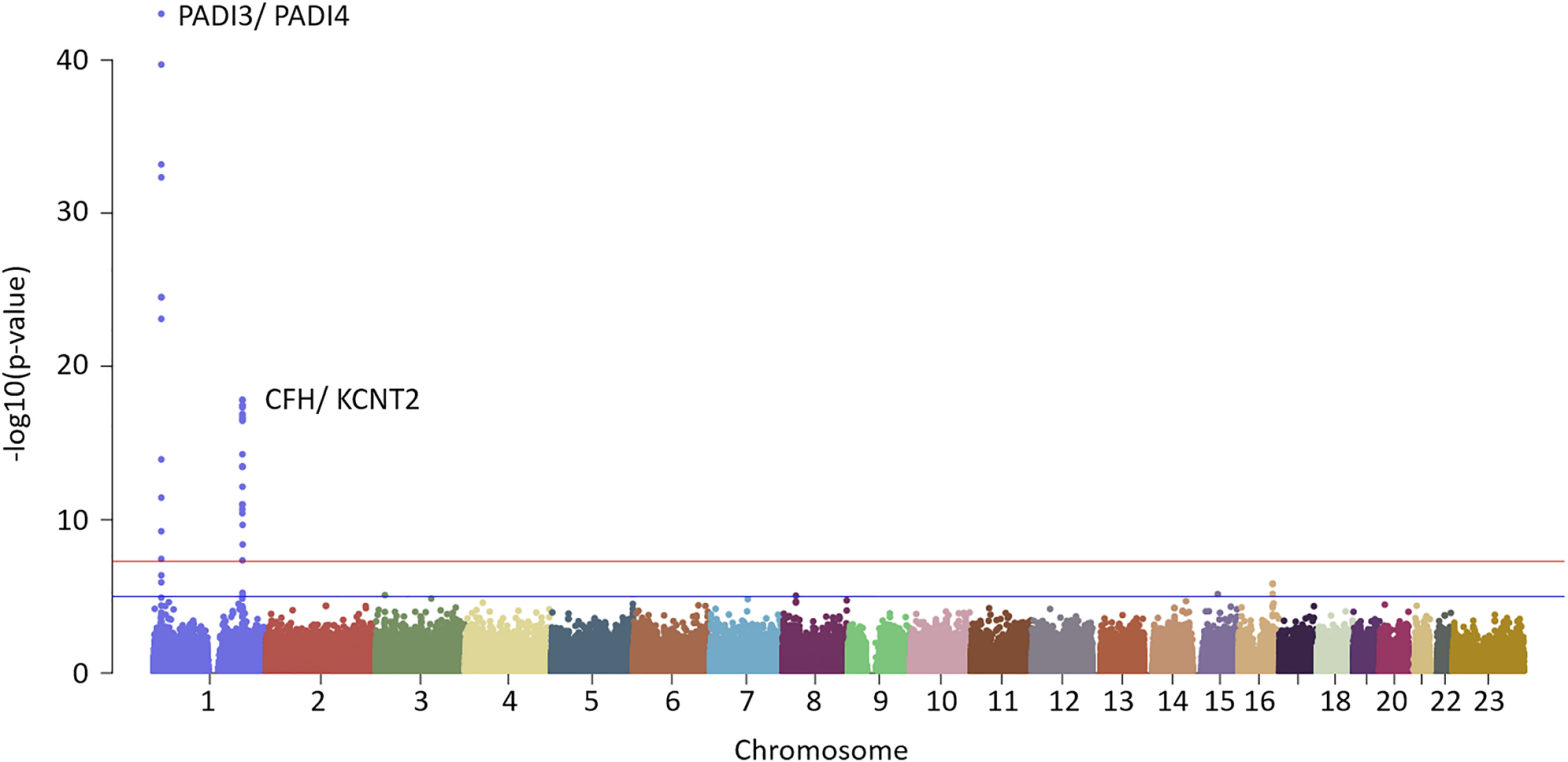
GWAS analysis identifies that VICM levels are associated with genetic variances in PADI3/ PADI4 and CFH/ KCNT2 loci. *PADI3/4* peptidylarginine deiminase 3/4, *CFH* complement factor H, *KCNT2* potassium sodium-activated channel subfamily T member 2.

GWAS was run to identify genetic polymorphisms associated with variation in serological VICM. The quantile–quantile plot did not show large deviation between observed and expected p-values (Supplementary Fig. S2). We identified 33 genome-wide significant associations with VICM, located on chromosome 1, in the PADI3/PADI4 locus, and the CFH/ KCNT2 locus. The resulting Manhattan plot from the analysis can be seen in Fig. 3. Clump analysis was performed to identify significantly associated SNPs which were in linkage disequilibrium. 11 genome-wide significant clumps were identified (Supplementary Table S4).

The most significant SNP identified, in the first clump, was rs13375202 (p = 9.81e−44, effect size = − 0.36 [95% CI − 0.38 to − 0.29]) located in the PADI4 locus. In the second most significant clump, we identified SNPs in the PADI3 gene, notably rs12037653 (p = 6.69e−34, effect size = − 0.28 [95% CI − 0.33 to − 0.24]). Whilst both SNPs have negative associations with VICM levels, other significant SNPs showed a positive correlation to VICM in the same locus. The SNP rs2240340 in the third most significant clump, in the PADI4 locus, showed significant correlation to VICM levels, whilst having a positive effect size (p = 3.08e−35, effect size = 0.24 [95% CI 0.20–0.29]).

Pathway enrichment analysis was performed using VEGAS2 and PARIS 2.4. The SNPs identified were significantly associated with pathways involved in the complement cascade as well as extracellular regulation. A full table of all associated pathways can be seen in Supplementary Table S5.

## Discussion

### Summary of findings

In this study we investigated genome-wide associations, and disease phenotypes with serological citrullinated and MMP-degraded vimentin (VICM). Through GWAS analysis of VICM, we observed 11 genome wide significant clumps explaining linkage disequilibrium between the 33 SNPs identified located in the PADI3/PADI4 locus and the CFH/ KCNT2 locus. Furthermore, we identified a statistically significant association with VICM and a history of chronic pulmonary disease as well as an increased risk of mortality from respiratory disease.

### PADI3/PADI4 association

We observed an association between VICM levels and SNPs in the PADI3/PADI4 region, with rs13375202 identified as the lead SNP. The PADI3/ PADI4 locus is a well-studied region, encoding the PAD3 and PAD4 enzymes, responsible for protein citrullination. Additionally, our finding also revealed that VICM levels were linked to chronic lung diseases and an increased rate of death due to respiratory diseases, aligning with previous studies. In a study by Lugli et al. elevated levels of the PAD4 protein in were found in lung tissue compared to control samples (spleen, skeletal muscle, liver, heart, kidney, lymph node, and ovary). Furthermore, patients with COPD exhibited higher PAD4 expression levels than their healthy counterparts, consistent with other studies reporting increased inflammation and citrullination in individuals with pulmonary diseases^1,11,23^. In a recent in vivo study citrullinated vimentin was also shown to mediate development and progression of lung fibrosis through TLR4-dependent NF-κB activation^8^. Citrullinated vimentin was sufficient to promote fibroblast activation in vitro and elicit profibrotic cytokine production and lung fibrosis in vivo, indicating that the PAD enzymes are a promising target to attenuate lung fibrosis.

Despite the well-known association between the more widely expressed PADI2 gene and vimentin citrullination, our study did not reveal a significant connection between VICM levels and SNPs in the PADI2 region. Further experiments are needed to understand the difference between the PAD enzymes in relation to citrullination of vimentin and development of lung fibrosis.

### CFH/ KCNT2 association

We also identified that VICM levels were associated with SNPs in the CFH/ KCNT2 region, with rs10801551 being the lead SNP. The CFH gene is responsible for the expression of complement factor H, which aids in the regulation of the body’s immune response through the complement system^24^. Previous studies have linked genetic changes in this gene to age-related macular degeneration^25^ and renal diseases^26,27^.

The compliment pathway has also, in previous studies, been shown to be upregulated in patient with IPF^28,29^. Furthermore, Gu et al. have shown that blocking the complement receptors C3aR and C5aR stops the progression of lung fibrosis and suppresses the complement action in vivo^30^. The complement system has a crucial role in innate immune response, and changes in the regulation can have profound consequences on the pathophysiology of patients, resulting in chronic inflammatory diseases^31^. This underlines the significance of this region as a strong target for therapeutic intervention or for monitoring disease development and activity.

### Clinical perspectives of VICM

The PADI3/ PADI4 and CFH/ KCNT2 loci are well studied regions, and currently used as targets for therapeutic intervention in auto-immune and inflammatory disease as well as some cancers^32,33^. The VICM biomarker may offer the possibility to measure some of the downstream processes associated with these regions, which could allow for monitoring of treatment response^34^. This has been previously shown by Mortensen et al. in a study of RA patients given mavrilimumab, an anti-GM-CSFRα-mAb drug^10^. It was observed that patients receiving treatment had significantly suppressed VICM levels when compared to controls. In addition, VICM was also demonstrated to be associated with macrophage activity in vitro*,* which is in line with the results presented in this study.

VICM may also be able to help predict disease progression or identify patients who will benefit most from treatments targeting citrullination and inflammatory processes. We have previously shown that VICM differed between endotypes of arthritic patients in a cluster analysis identifying patients with faster disease progression^35^. Together with the results of this study these findings suggest that VICM may be used as a disease activity marker measuring both citrullination and inflammation. By combining VICM with other markers of specific tissue turnover, better patient profiles can potentially be identified for combination therapy and to identify patients with active diseases.

### Limitations

The PERF cohort consist of only post-menopausal Danish women. The results of this paper may therefore not be applicable to other age-groups, nationalities, and genders. Furthermore, due to the nature of the cohort being observational, it may be possible that some diseases have been underrepresented, as we did not have access to data from general physicians. Biomarker levels may also have been modulated due to pharmaceutical intervention which we were not able to control for. For the SNP-disease associations reported in this study, the effect size is relatively small. This may reflect the complexity of the diseases which have many factors affecting their onset, whilst it may also reflect the heterogeneity of the PERF population, which has not been designed for these tasks. For many disease phenotypes, the analysis was largely underpowered due to population size. It is possible that in a larger population, more biomarker-genotype and biomarker-disease associations would have been found.

## Conclusion

We observed that serum VICM levels was associated with chronic pulmonary diseases and SNPs in loci related to citrullination and immune regulation (PADI3/PADI4 and CFH/ KCNT2). This points to the potential for VICM to be used as an activity marker of both citrullination and inflammation identifying responders to treatment and patients likely to experience disease progression.

### Supplementary Information

Supplementary Information.

## Data Availability

The datasets used and/or analyzed during the current study available from the corresponding author on reasonable request.

## Abbreviations

ACPA: Anti-citrullinated protein antibodies
CFH: Complement factor H
COPD: Chronic obstructive pulmonary disease
CPR number: Civil registration number
CRP: C-reactive protein
CVD: Cardiovascular disease
ELISA: Enzyme-linked immunosorbent assay
GWAS: Genome wide association study
IBD: Inflammatory bowel disease
ICH-GCP: International conference on harmonization—guideline for good clinical practice
IPF: Idiopathic pulmonary disease
MMP: Matrix metalloproteinase
NLR: Neutrophil–lymphocyte ratio
NF-κB: Nuclear factor kappa B
NPR: Danish national patient registry
PAD: Peptidylarginine deiminase
PADI3: Peptidylarginine deiminase 3
PADI4: Peptidylarginine deiminase 4
PERF: Prospective epidemiologic risk factor study
PheWAS: Phenome wide association study
RA: Rheumatoid arthritis
SNP: Single nucleotide polymorphisms
TLR4: Toll-like receptor 4
VICM: Citrullinated and MMP-degraded vimentin
