# Supplementary material for: Citrullinated and MMP-degraded vimentin is associated with chronic pulmonary diseases and genetic variants in PADI3/PADI4 and CFH in postmenopausal women
Source: Sci Rep. 2023 Dec 27;13:23039. doi: 10.1038/s41598-023-50313-y (PMC10754934; doi:10.1038/s41598-023-50313-y)
Supplement: Supplementary file 1 — Supplementary Information. [file 41598_2023_50313_MOESM1_ESM.docx]

# Supplementary Materials

**Supplementary Table 1. Overview of the disease comorbidities present in the population at baseline and end of study for patients in the PERF cohort**

| Disease Phenotypes | Baseline | End of study |
| --- | --- | --- |
| Alzheimer's Dementia (%) | 81 (1.9) | 626 (14.3) |
| Hyperlipidemia (%) | 918 (21.0) | 1257 (28.8) |
| Diabetes (%) | 292 (6.7) | 720 (16.5) |
| Hypertension (%) | 1638 (37.5) | 2604 (59.6) |
| Ischemic Heart Disease (%) | 224 (5.1) | 698 (16.0) |
| Peripheral Arterial Disease (%) | 330 (7.6) | 477 (10.9) |
| Congestive Heart Failure (%) | 79 (1.8) | 512 (11.7) |
| Rheumatic Disease (%) | 199 (4.6) | 421 (9.6) |
| Osteoarthritis (%) | 1507 (34.5) | 2278 (52.1) |
| Inflammatory Bowel Disease (%) | 61 (1.4) | 202 (4.6) |
| Chronic Pulmonary Disease (%) | 243 (5.6) | 938 (21.5) |
| Gastrointestinal Cancer (%) | 74 (1.7) | 244 (7.9) |
| Respiratory Cancer (%) | 19 (0.4) | 142 (3.3) |
| Breast Cancer (%) | 149 (3.4) | 323 (7.4) |
| Chronic Kidney Disease (%) | 824 (18.9) | 1078 (24.7) |
| Chronic Liver Disease (%) | 96 (2.2) | 170 (3.9) |
| osteoporosis (%) | 1014 (23.2) | 1513 (34.6) |
| Cerebrovascular Disease (%) | 225 (5.1) | (21.7) |

**Supplementary Table 2. Disease Phenotype Definitions used in the PERF cohort**

| **Clinical Phenotype** | **Definition** |
| --- | --- |
| Hyperlipidemia | Hospitalization or cause of death due to ICD10 codes E780-E785; **or** Serum cholesterol levels > 7.5mmol/L; **or** Answered yes to questions regarding ‘Hyperlipidemia’ in a medical history questionnaire; |
| Diabetes | Hospitalization or cause of death due to ICD10 codes E10-E14, O24, H360; **or** Serum glucose level ≥ 7mmol/L; **or** Blood B-A1C measures ≥ 48mmol/mol**; or** Had been added to the National Diabetes Registry; **or** Answered yes to questions regarding ‘Diabetes’ in a medical history questionnaire; **or** Has a record in the National Treatment Registry BBHA for diabetes treatment |
| Hypertension | Hospitalization or cause of death due to ICD10 codes I10-I15; **or** diastolic blood pressure **and** systolic blood pressure over 90 and 140 respectively; **or** Answered yes to questions regarding ‘Hypertension’ in a medical history questionnaire; |
| Alzheimer’s + Dementia | Hospitalization or cause of death due to ICD10 codes F00-F03, G30-G32 or R54; **or** Short Blessed Test Score ≥ 10 **and** ART score ≤ 14 respectively; **or** Answered yes to questions regarding ‘Alzheimer’s Disease’ in a medical history questionnaire; |
| Ischemic Heart Disease (Coronary Artery Disease) | Hospitalization or cause of death due to ICD10 codes I21-I25; **or** Answered yes to questions regarding ‘Ischemic Heart Disease’ in a medical history questionnaire; **or** A record in the National Danish Operation Registry with at least one OPR code from KFNC, KFND, KFNE, KFNG |
| Peripheral Arterial Disease | Hospitalization or cause of death due to ICD10 codes I702, I739; **or** Answered yes to questions regarding ‘Peripheral Arterial Disease’ in a medical history questionnaire; |
| Congestive Heart Failure | Hospitalization or cause of death due to ICD10 codes I099, I110, I130, I132, I255, I420, I425, I429, I50, I1971A, O291A, O742A, P290; **or** Answered yes to questions regarding ‘Heart Failure’ in a medical history questionnaire |
| Rheumatic Disease (RA) | Hospitalization or cause of death due to ICD10 codes M05, M06, M08, M315, M32-M34, M351, M353, M360, M45, M461, M468, M469; **or** Answered yes to questions regarding ‘Rheumatic Disease’ in a medical history questionnaire; **or** A record in the National Danish Treatment Registry with a OPR code BOHJ18 |
| Osteoarthritis | Hospitalization or cause of death due to ICD10 codes M15-M19; **or** Answered yes to questions regarding ‘Osteoarthritis’ in a medical history questionnaire **or** A record in the National Danish Operation Registry with at least one OPR code from KNFB, KNGB |
| IBD (Crohn’s and Colitis) | Hospitalization or cause of death due to ICD10 codes K50-K52; **or** Answered yes to questions regarding IBD in a medical history questionnaire; |
| Chronic Pulmonary Disease | Hospitalization or cause of death due to ICD10 codes I278, I279. J40-J47, J60-J67, J684, J701, J703, J84, J96; **or** Answered yes to questions regarding Chronic Pulmonary Disease in a medical history questionnaire; **or** A record in the National Danish Treatment Registry with a code BJKC, BGD; **or** A record in the National Danish Operation Registry with a OPR code KGDG |
| Gastrointestinal Cancer | Hospitalization or cause of death due to ICD10 codes DC15-DC26, Z080D, Z080G, Z081D, Z081G, Z082D, Z082G, Z087, Z850; **or** Answered yes to questions regarding Gastrointestinal Cancer in a medical history questionnaire |
| Chronic Liver Disease | Hospitalization or cause of death due to ICD10 codes I85, K70, K72-K74, K752-K759, K76, K77, Q446, R160, B942, P788A, B180-B182 ; **or** A record in the National Danish Operation Registry with a OPR code KPHW35A, KPHW45-47, KJJA20-26; **or** A record in the National Danish Treatment Registry with code BUBC3, BIKH;  **or** Answered yes to questions regarding Liver Disease in a medical history questionnaire |
| Respiratory Cancer | Hospitalization or cause of death due to ICD10 codes C30-C39, Z08B, C80W, Z81B, Z81W, Z82B, Z82W, Z087B, Z87W, Z851; **or** Answered yes to questions regarding Respiratory Cancer in a medical history questionnaire |
| Breast Cancer | Hospitalization or cause of death due to ICD10 codes C50, Z080R, Z081R, Z0802R, Z087R, Z853; **or** Answered yes to questions regarding Breast Cancer in a medical history questionnaire; **or** A record in the National Danish Operation Registry with a OPR code KHAF |
| Chronic Kidney Disease | Hospitalization or cause of death due to ICD10 codes A1811, B520, D593, E102, E112, E122, E132, E142, E853B, I12, I13, I151, K767, M103, N02, DN03, DN04, N05, N06, N07, N08, N131, N132, N133, N134, N135, N136, N137, N138, N139, N15, N16, N18, N19, N25, N26, N289, O0904, O084, O102, O103, Q61, Q62, Q63, R944, T855A1, T856C, T817Y4, T855A1, Z087H, Z905B, Z940, Z992; **or** A record in the National Danish Operation Registry with a OPR code KKAB, KKAC, KKAD, KKAE, KKAF00-01, KKAH, KKAS, KKAT; **or** A record in the National Danish Treatment Registry with code BJFD, BJHA, BJFZ, BJKB, BUBA, BWCD5; **or** eGFR score < 60; **or** Reported ‘renal or kidney impairment’ in a medical history questionnaire |
| Ovarian cancer | Hospitalization or cause of death due to ICD10 codes C51-C56, DD069; **or** Answered yes to questions regarding Ovarian Cancer in a medical history questionnaire; **or** Reported ‘ovarian cancer’ in medical history questionnaire |
| Atrial fibrillation | Hospitalization or cause of death due to ICD10 codes I48 |
| Cerebrovascular disease | Hospitalization or cause of death due to ICD10 codes I60, I61, I62, I63, I64, I65, I66, I67, I68, I69, G45 **or** Answered yes to a question regarding ‘Cerebrovascular Disease’ in a medical history questionnaire |
| Osteoporosis | Hospitalization or cause of death due to ICD10 codes M80, M81, M82 **or** Answered yes to a question regarding ‘Osteoporosis’ in a medical history questionnaire **or** had a T-score ≤ -2.5 |

**Supplementary Table 3. Hazard of death due to specific causes with an increase of VICM by a factor of 2.**

| Cause of mortality (n) | Hazard (confidence interval) | | p-value |
| --- | --- | --- | --- |
|  |  | |  |
| All Cause (1437) | 1.09 (1.03-1.18) | 0.007 | |
| Cancer (419) | 1.01 (0.89-1.15) | 0.76 | |
| Respiratory Disease (192) | 1.27 (1.06-1.52) | 0.009 | |
| Cardiovascular Disease (490) | 1.21 (1.08-1.36) | 0.001 | |

**Supplementary Table 4. SNP Clumps identified through GWAS analysis of serological VICM in the PERF cohort**

| Chromosome | | Representative SNP | Base pair | P-value | Clump Members |
| --- | --- | --- | --- | --- | --- |
| 1 | rs13375202 | | 17325142 | 9.82e-44 | rs2240335 |
| 1 | rs12037653 | | 17273822 | 6.69e-34 | rs2293917, rs2501784 |
| 1 | rs2240340 | | 17336144 | 3.08e-25 | rs1748041, GSA-rs11203366 |
| 1 | GSA-rs10801551 | | 196610909 | 1.45e-18 | rs946750, rs10922084, rs28582999, rs12029571, rs7524776, rs514591, rs800292 |
| 1 | rs7531555 | | 196960180 | 3.26e-18 | rs12116643, rs10922162 |
| 1 | rs395544 | | 196729142 | 1.2e-17 | rs1329424, rs1061147, rs10801555, rs1831282, rs1410996, rs380390, rs1329428 |
| 1 | rs7548900 | | 17296159 | 1.13e-14 | None |
| 1 | rs6695321 | | 196706731 | 6.9e-13 | None |
| 1 | GSA-rs2477131 | | 17309826 | 3.55e-12 | None |
| 1 | rs6682225 | | 196463028 | 2.05e-11 | rs1538686 |
| 1 | GSA-rs2977295 | | 17273942 | 3.55e-08 | None |

**Supplementary Table 5. Pathways significantly associated to SNPs identified from GWAS, found using VEGAS and PARIS pipelines**

| Reactome-ID | Description | PARIS empirical P-value | VEGAS empirical P-value |
| --- | --- | --- | --- |
| R-HSA-159227 | Transport of the SLBP independent Mature mRNA | 0.002 | 0.0454 |
| R-HSA-159231 | Transport of Mature mRNA Derived from an Intronless Transcript | < 0.001 | 0.034 |
| R-HSA-165054 | Rev-mediated nuclear export of HIV RNA | < 0.001 | 0.050 |
| R-HSA-168271 | Transport of Ribonucleoproteins into the Host Nucleus | 0.004 | 0.048 |
| R-HSA-168333 | NEP/NS2 Interacts with the Cellular Export Machinery | 0.002 | 0.027 |
| R-HSA-170822 | Regulation of Glucokinase by Glucokinase Regulatory Protein | 0.001 | 0.044 |
| R-HSA-180746 | Nuclear import of Rev protein | 0.002 | 0.029 |
| R-HSA-180910 | Vpr-mediated nuclear import of PICs | 0.001 | 0.030 |
| R-HSA-189200 | Cellular hexose transport | 0.015 | 0.0026 |
| R-HSA-3108214 | SUMOylation of DNA damage response and repair proteins | 0.019 | 0.028 |
| R-HSA-373076 | Class A/1 (Rhodopsin-like receptors) | 0.002 | 0.034 |
| R-HSA-432722 | Golgi Associated Vesicle Biogenesis | < 0.001 | 0.024 |
| R-HSA-446353 | Cell-extracellular matrix interactions | < 0.001 | 0.029 |
| R-HSA-4570464 | SUMOylation of RNA binding proteins | < 0.001 | 0.030 |
| R-HSA-5578749 | Transcriptional regulation by small RNAs | 0.003 | 0.044 |
| R-HSA-6783310 | Fanconi Anemia Pathway | 0.005 | 0.012 |
| R-HSA-977606 | Regulation of Complement cascade | < 0.001 | 0.00068 |

**
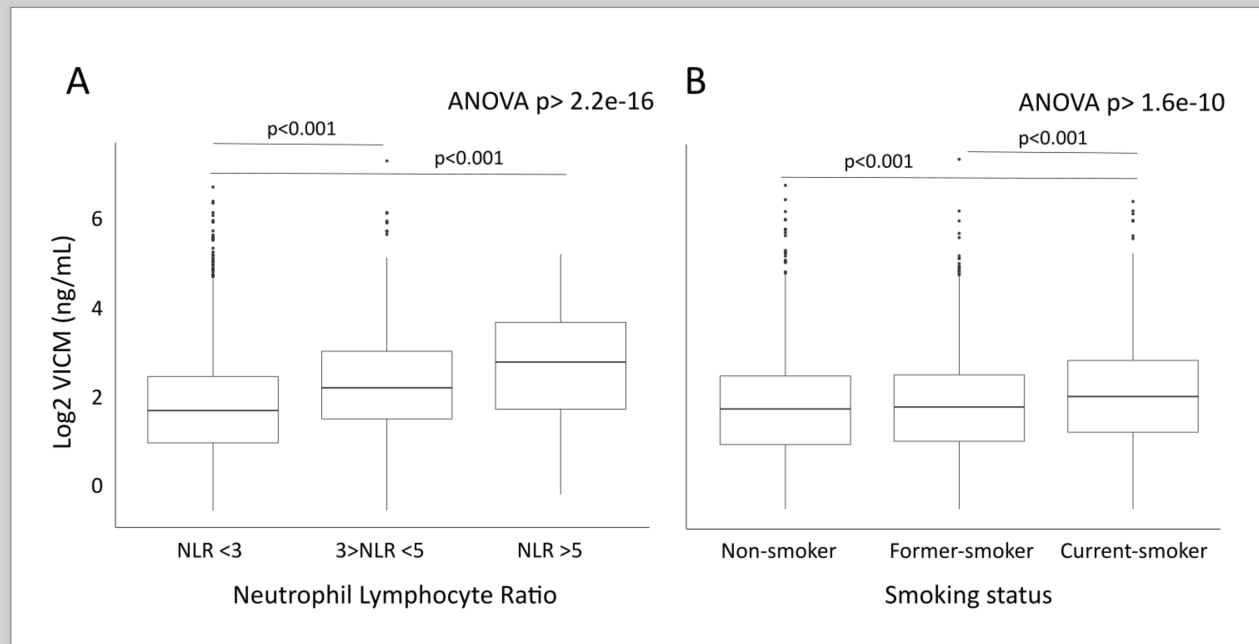
**

**Supplementary Figure 1: VICM biomarker levels are significantly associated with neutrophil to lymphocyte ratio (NLR) (A) and smoking status (B).**


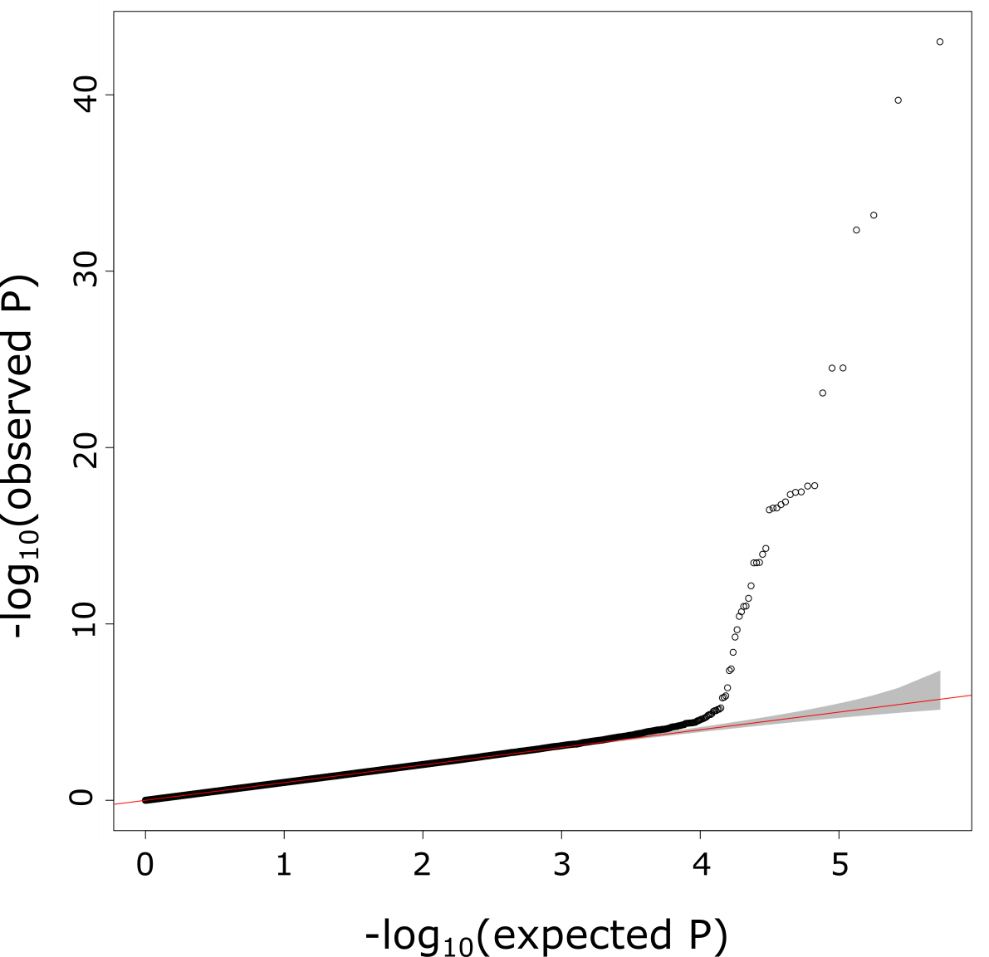


**Supplementary Figure 2. Quantile-Quantile (QQ) plot of the data shown in the Manhattan plot**
